# Attentional Control and Subjective Executive Function in Treatment-Naive Adults with Attention Deficit Hyperactivity Disorder

**DOI:** 10.1371/journal.pone.0115227

**Published:** 2014-12-29

**Authors:** Venke Arntsberg Grane, Tor Endestad, Arnfrid Farbu Pinto, Anne-Kristin Solbakk

**Affiliations:** 1 Department of Neuropsychology, Helgeland Hospital, Mosjøen, Norway; 2 Department of Psychology, University of Oslo, Oslo, Norway; 3 Department of Neurosurgery, Division of Surgery and Clinical Neuroscience, Oslo University Hospital—Rikshospitalet, Oslo, Norway; Georgetown University, United States of America

## Abstract

We investigated performance-derived measures of executive control, and their relationship with self- and informant reported executive functions in everyday life, in treatment-naive adults with newly diagnosed Attention Deficit Hyperactivity Disorder (ADHD; n = 36) and in healthy controls (n = 35). Sustained attentional control and response inhibition were examined with the Test of Variables of Attention (T.O.V.A.). Delayed responses, increased reaction time variability, and higher omission error rate to Go signals in ADHD patients relative to controls indicated fluctuating levels of attention in the patients. Furthermore, an increment in NoGo commission errors when Go stimuli increased relative to NoGo stimuli suggests reduced inhibition of task-irrelevant stimuli in conditions demanding frequent responding. The ADHD group reported significantly more cognitive and behavioral executive problems than the control group on the Behavior Rating Inventory of Executive Function-Adult Version (BRIEF-A). There were overall not strong associations between task performance and ratings of everyday executive function. However, for the ADHD group, T.O.V.A. omission errors predicted self-reported difficulties on the Organization of Materials scale, and commission errors predicted informant reported difficulties on the same scale. Although ADHD patients endorsed more symptoms of depression and anxiety on the Achenbach System of Empirically Based Assessment (ASEBA) than controls, ASEBA scores were not significantly associated with T.O.V.A. performance scores. Altogether, the results indicate multifaceted alteration of attentional control in adult ADHD, and accompanying subjective difficulties with several aspects of executive function in everyday living. The relationships between the two sets of data were modest, indicating that the measures represent non-redundant features of adult ADHD.

## Introduction

Attention Deficit Hyperactivity Disorder (ADHD) is a common early-onset neurodevelopmental syndrome with an estimated prevalence of 2.2% for males and 0.7% for females [1]. It is characterized by the principal symptoms inattention, hyperactivity, and impulsivity [2]. The precise neurobiological basis of ADHD remains unclear. Studies suggest involvement of multiple neural systems, but atypical development of frontal-striatal circuitry and associated dopaminergic pathways is commonly emphasized [3], [4]. ADHD has traditionally been considered a childhood disorder, but there is accumulating evidence for the validity of the diagnosis in adulthood [5]–[11].

The recent clinical understanding that ADHD may persist into adulthood has resulted in an increase in referrals of adults without a childhood diagnosis. This poses challenges of diagnosis as it is difficult retrospectively to obtain accurate information on childhood behavior, and because the diagnostic criteria give limited consideration to the heterogeneity of the disorder and its developmental course [12], [13]. Compared with childhood ADHD, there is still limited empirical knowledge about clinical features and neurocognitive function in adult ADHD [14]–[16].

The manifest symptoms of ADHD may change over time [17]. Adults with persisting symptoms tend to report difficulties related to executive function, including controlled attention, more often than behavioral hyperactivity and impulsivity [18]. In a qualitative review of neuropsychological studies, Woods and colleagues found that the majority showed significant impairment on at least one measure of executive function. The most reliable tests were those demanding selective and sustained attention, and motor response inhibition [19]. Later empirical [14], [20] studies and meta-analytic reviews [21]–[23] have supported that adults with ADHD tend to have particularly low scores on tests requiring different aspect of controlled attention, although deficits in non-executive cognitive domains such as information processing and motor speed have also been observed. Importantly, a cumulative performance decrement resulting from increases in task demands related to complexity, time requirements, processing speed, and motor functions has been noted [22]. Thus, early executive control deficits in ADHD appear to persist into adulthood [15], and might be consistent with self- and informant reports regarding behavioral manifestations of executive difficulties in everyday life [24]–[26].

Because attention is a basic, but multifaceted function upon which all neurocognitive processes rely, dysfunction of components of attention may partly underlie difficulties with complex executive function such as planning, problem solving, and decision-making. A well-known clinical observation is that persons with ADHD can engage sustained attentional effort when highly motivated for a task or activity [25], [26]. Thus, inattention is not an invariant feature of ADHD, but changes with motivational factors and environmental requirements for cognitive control. Leading an independent and productive adult life requires rapid and flexible adaptation of behavior according to changing external circumstances. This is typically denoted executive function and demands a high degree of attentional and behavioral control, which seems to be compromised in adult ADHD [24], [27].

In order to better understand the neurocognitive and behavioral executive deficits associated with adult ADHD there is a need for more detailed characterization of dysfunctions and the conditions modulating their expression. The objective of the present study was to examine attentional and behavioral control in treatment-naive adults with newly diagnosed ADHD, using a task paradigm which requires sustained attention and entails experimental manipulation of important parameters of attention and response control. A secondary aim was to explore whether cognitive performance on a laboratory-based task of sustained attention is associated with subjective reports of executive function.

Although deficits in sustained and selective attention are thought to influence modern living skills, such as time management, driving a car in complex urban environments, or using technically advanced communication media, their importance for complex cognitive functions and behavior are infrequently addressed [28].

We employed the Test of Variables of Attention (T.O.V.A.) [29] which belongs to the family of Continuous Performance Tests (CPTs), found to be useful for assessing core aspects of attentional and behavioral control in clinical populations. Although impaired performance is not specific to ADHD, CPTs are highly sensitive to ADHD in children and adolescents [30]–[33], as well as in adults [34]–[37].

Importantly, a study by Advokat and coworkers showed that adults with ADHD differed from non-clinical adults and adults with psychiatric disorders, but not adults with learning disorders, on errors of omission, reaction time, and reaction time variability in the Conner's CPT [38]. The T.O.V.A. adheres to current opinion that attention is a multidimensional function and can be divided into different sub-functions which are mediated by partly separate neural networks [39]–[42]. It allows assessment of behavioral indices of information processing speed, response accuracy, and inhibition of prepotent responses, all of which have been found to be impaired in childhood ADHD [43], [44]. To our knowledge, the number of adult ADHD studies that included a healthy control group for comparison of performance on the T.O.V.A. is sparse. Weyandt et al. reported that adults (primarily college students) with ADHD made more omission errors on the T.O.V.A. compared to college students without ADHD [45]. The T.O.V.A. has been used in other studies on adult ADHD, but these employed within-group designs to examine change in T.O.V.A. performance and did not include healthy and demographically matched control groups to determine whether performance in the ADHD group was impaired at baseline [46]–[48].

Based on the extant literature on cognitive difficulties in adult ADHD [3], [14], [20], [27], [38], [46], we expected that mechanisms of attention and accompanying motor response control would be adversely affected in adult ADHD, and that deficits would be particularly evident in task conditions demanding a high degree of attentional control.

We hypothesised that impaired sustained attention in our cohort of adult ADHD patients would be reflected in an increased rate of Go signal omission errors and variable response speed to detected Go signals in the T.O.V.A. Furthermore, we expected that impaired inhibitory motor control, as indexed by the rate of NoGo commission errors, would be particularly pronounced in task conditions with a high strength of response prepotency, such as when the frequency of Go signals increases relative to NoGo signals.

In light of the long-standing concern about the ecological validity of laboratory-derived measures of executive control [5], [47], [49], we also examined whether there were associations between T.O.V.A. performance parameters and reported executive difficulties in daily life. For this purpose, we correlated T.O.V.A. performance scores with scores on the Behavior Rating Inventory of Executive Function-Adult Version (BRIEF-A) [48]. The BRIEF-A is a questionnaire designed for self- as well as informant-report concerning diverse areas of cognitive and behavioral executive functioning [48]. As task performance and ratings of everyday behaviors are measures at different levels of analysis, and provide partly different windows into brain function, we did not expect strong relationships between them [50]. The BRIEF-A has been shown to be useful in identifying clinically meaningful executive problems that may not be detected with conventional neuropsychological tests of executive function [50], [51]. Due to accruing evidence that performance on tasks considered void of emotionally charged content can be influenced by emotional states [52], [53], and because adult ADHD is associated with inceased risk of coexisting psychophatology such as depressed mood and anxiety [53], [54], we also explored the relationship between scores on the T.O.V.A. and self-reported symptoms of low mood and anxiety on the Adult-Self-Report (ASR) of the Achenbach System of Empirical Based Assessment (ASEBA) [55].

## Methods

### Recruitment and participants

Patients with ADHD (n = 36; mean age  = 31.8 years; age range  = 19–53 years; mean education  = 11.0 years; education range  = 10–16 years) were recruited from a consecutive series of adult patients referred to the Department of Neuropsychology, Helgeland Hospital, in the time period from 2008 to 2011. All patients were referred from specialist mental health services or primary care physicians for a second opinion evaluation of suspected ADHD.

A comparison group of 35 healthy controls were recruited from three medium-size companies in the Helgeland area to match the patient group on the variables age, years of completed education, and sex (mean age  = 32.2 years; age range  = 18-51 years; mean education  = 11.5years; education range  = 10–17 years). From lists of all employees, those who fulfilled the matching criteria for a patient were contacted via telephone and asked to participate in the study. A random draw was conducted in cases where more than one employee fulfilled the matching criteria.

All participants were questioned about their past and present somatic and psychological health, as well as completed education. Criteria for exclusion were: a) history of mild to severe traumatic brain injury, b) neurological disorder, c) history of severe memory problems, d) diabetes, e) metabolic disorder, f) psychotic symptoms, g) alcohol and/or substance abuse requiring treatment, and h) visual or auditory sensory loss.

Inclusion in the ADHD group was based on the DSM-IV criteria for ADHD [56] assessed by a senior neuropsychologist during a semi-structured interview (Adult Interview) [57]. The interview comprises assessment of current and childhood DSM-IV-defined ADHD symptoms and related impairment in the school/work and home setting, including social relationships. Past and present psychiatric comorbidities were assessed. Participants were also questioned about learning problems and psychiatric illness in close family members. All patients included in the study met the DSM-IV criteria for ADHD, combined type, as they had significant behavioral impairment related to both inattention and hyperactivity/impulsivity. None of the healthy controls met the DSM-IV criteria for ADHD. Six patients were not included in the study because they did not meet the DSM-IV diagnostic criteria for ADHD, and/or were rejected based on other exclusion criteria.

The Adult Self-Report (ASR) of the Achenbach System of Empirically Based Assessment (ASEBA) was completed by all participants in order to obtain an independent ADHD score, status in relation to work or study, as well as neuropsychiatric comorbidity [55]. At the time of making a diagnosis, the results from the ASR, T.O.V.A., and the BRIEF-A were not known to the clinical neuropsychologist, and hence not included in the diagnostic assessment.

The patient and healthy control samples were of Western European descent, thus representative of the ethnic composition of the region. All participants had Norwegian as first language. They were right-handed, except for one ADHD patient and one healthy control who were left-handed. Prior to inclusion in the study, all participants were tested for auditory (audiometry test) and vision (optometry test) deficits. They had either normal acuity or vision corrected by optical lenses. All had normal hearing. None had received stimulant medications prior to or during participation in the study. The general IQ of all participants was assessed with the full version of the Wechsler Adult Intelligence Scale, 3^rd^ edition (WAIS–III) [58]. The patient and healthy control groups did not differ significantly in age, sex, years of completed education, or total IQ (Table 1).

**Table 1:** Participant demographics and T-scores on the ASEBA Adult-Self-Report (ASR) DSM ADHD Problems scale.

	ADHD (n = 36)		Control (n = 35)		t-test/chi square
Variable	Mean	SD	Mean	SD	p
Age (years)	31.8	10.0	32.2	9.5	.885
Male/Female	17/19		17/18		.911
Education completed	10.4	1.9	10.9	2.3	.301
Work or study (%)	69		100		
Total IQ (WAIS III)	93.8	13.1	99.1	10.8	.067
ASR DSM ADHD	75.8	11.0	52.0	3.1	.001

Group means and standard deviations (SD) are shown. Notes. Education  =  total years of education completed; Work or study  =  percentage of participants working or studying during the last 6 months; ns  =  non-significant.

### Ethics Statement

All participants gave written, informed consent to take part in the study. The study was approved by the Regional Committee for Medical and Health Research Ethics – North Norway, and was conducted in agreement with the Helsinki Declaration.

### Neuropsychological test paradigm—Test of Variables of Attention

**Figure 1:**
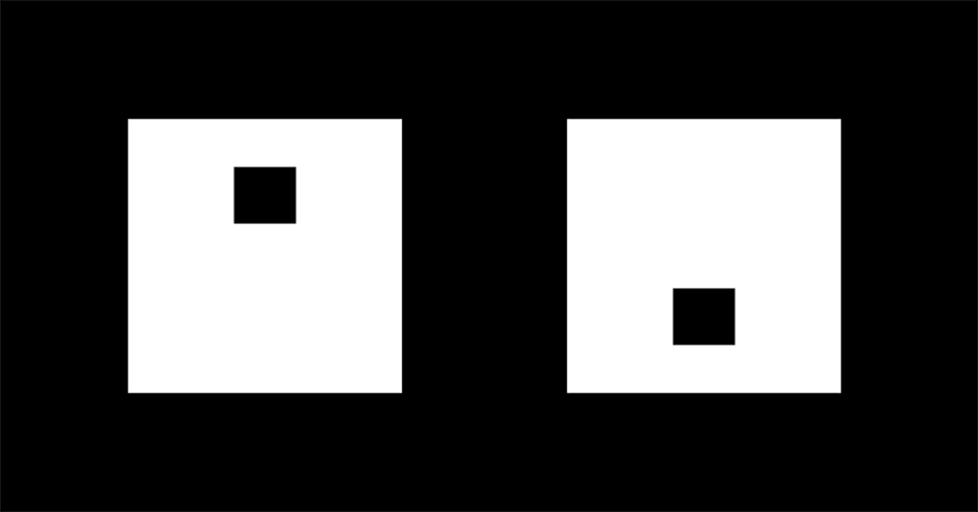
Go (left) and NoGo (right) signals in the T.O.V.A. Reprinted with permission from the TOVA Company, Los Alamitos, CA 90720.

The T.O.V.A. (version 7.3) [29] is a computer-administered CPT paradigm developed to measure sustained attention and impulse control. It lasts for 21.6 minutes, which is longer than patients suffering from ADHD can typically stay vigilant, and may therefore be more sensitive to problems with sustained attention than CPTs of shorter duration [59], [60]. It has a non-language-based format that requires no left-right discrimination or sequencing. In the visual task the stimuli are two easily discriminated geometric figures centered on the computer screen (Fig. 1). The use of geometric stimuli avoids possible confounding effects of letter discrimination problems related to potential comorbid reading disorder. The T.O.V.A. requires the person to maintain vigilance while responding continuously to target (Go) signals and inhibiting responses to infrequent nontarget (NoGo) signals that briefly flash on a computer screen [61]. The task includes two different conditions. The first half, containing infrequent Go signals, is known to be sensitive to problems of inattention, whereas the second half, containing frequent Go signals, is useful to detect problems with impulse control. The differentiated task demand allows one to evaluate if the person can adjust effectively to a new task condition.

One of the two geometric figures is presented for 100 milliseconds every 2 seconds. The designated Go signal is presented 22.5% (n = 72) of the trials during Condition 1 (Go signal infrequent condition) which constitutes the 1^st^ half of the task (comprising quarters 1 and 2), and 77.5% (n = 252) of the trials during Condition 2 (Go signal frequent condition) which represents the 2^nd^ half (comprising quarters 3 and 4) of the task. The stimuli are presented in a fixed random frequency per quarter. The task is to respond as quickly as possible to the Go signals (a small hole near the top of the square) by pressing a microswitch, and to inhibit responding to the GoNo signals (a small hole near the bottom of the square). The varying Go/NoGo ratio allows examination of the effects of differing response demands on performance.

The T.O.V.A. software program precisely records all responses, nonresponses, and response times. We analyzed the primary performance parameters Go signal reaction time and reaction time variability, Go signal omission errors, and NoGo signal commission errors for each quarter, half, and total of the task.

*Reaction time* is the measure of time taken to respond correctly, from the onset of the Go signal until the microswitch (having millisecond precision) is pressed. The reaction time score is the average of the correct Go response times, in which the sum of all correct reaction times is divided by the number of Go stimuli and is calculated in milliseconds. The formula used is as follows: Reaction time  =  ∑ Correct Response Times/# targets.

*Reaction time variability* is the participant's reaction time variance or inconsistency in reaction times. The score is reported as the standard deviation of the mean correct Go signal reaction times; i.e., Reaction time variability  =  (∑ Response Times – Mean Correct Response Time)^2^/# Correct Responses.

*Omission errors* are recorded when participants do not respond to the Go signals, and are measured as a ratio of the participant's correct responses to Go signals to the actual number of Go signals presented minus the number of anticipatory responses to Go signals: Omission errors  =  # Omissions/# Go stimuli - # Go stimuli Anticipatory Responses. Anticipatory responses are measures of guessing which stimulus will or will not be presented, and is also explained as a strategy to react to any stimulus as soon as possible [29], [62]. Anticipatory responses are recorded when the subject presses the microswitch between 200 milliseconds before and after the appearance of any stimulus (Go or NoGo).

*Commission errors* are recorded when the participant fails to inhibit responding and incorrectly presses the button to NoGo signals. The score is measured as a ratio of the incorrect responses to NoGo signals to the actual number of NoGo signals presented minus the number of anticipatory responses to NoGo signals: Commission Errors  =  (# Commissions/# NoGo stimuli - # NoGo stimuli Anticipatory Responses) X 100.

#### Validity of individual T.O.V.A. performances

All individual T.O.V.A. protocols were evaluated with regard to validity in accordance with the recommendations and criteria provided in the T.O.V.A. 7 Symptoms Exaggeration Index (SEI) Worksheet (http://www.tovatest.com/clinicalsupport/TOVA_SEI_worksheet.pdf). The SEI is based on the presence of response patterns that are not characteristic of clinical disorder and by lack of internal consistency in reaction time scores across specific response types. We employed a cut-off criteria for exclusion of SEI  = 3, indicating strong evidence of possible symptoms exaggeration. None of the participants had a SEI score of 3. Thirty-two patients and 34 controls had no evidence of symptom exaggeration (Total SEI score  = 0–1). One healthy control and 4 ADHD patients had some evidence of possible symptom exaggeration (Total SEI score  = 2). We included these patients for further analyses as other assessment, including validity of BRIEF-A self-report and clinical interview, gave no evidence of compromised validity.

### Behavior Rating Inventory of Executive Function-Adult Version

Assessment of executive problems in everyday living was done by means of self- and informant report on a Norwegian translation of the BRIEF-A [48]. The BRIEF-A is a standardized rating scale designed to evaluate everyday behaviors related to specific domains of executive function in adults. It consists of 75 items reflecting behaviors to be rated as often, sometimes or never being a problem during the past 4 weeks. These form 9 nonoverlapping, theoretically and statistically derived scales than in turn make up 2 summary index scales. The Metacognition Index is composed of 5 scales: Initiate, Working Memory, Plan/Organize, Task Monitor, and Organization of Materials, whereas the Behavioral Regulation Index consists of 4 scales: Inhibit, Shift, Emotional Control, and Self-Monitor. The additional Global Executive Composite scale summary measure incorporates all of the clinical scales and reflects overall executive functioning. Standard scores are computed for each of the clinical scales, indices, and for the summary composite. The reported T scores for each group of respondents are based on comparison to the normative sample of 1050 self- and 1200 informant reports. Higher scores reflect greater difficulties, with T scores ≥65 considered clinically significant. The BRIEF-A also includes 3 validity scales indicating whether respondents tend to have an unusually negative response style (Negativity scale), report highly unusual symptoms (Infrequency scale), or tend to answer similar items in an inconsistent manner (Inconsistency scale). The patients and the healthy controls completed the self-report version, whereas a close relative of the patients filled out the informant version of the BRIEF-A. There were missing self-report data from 1 patient and 3 controls, and from 2 patient informants.

Validity of individual BRIEF-A protocols. All individual BRIEF-A reports were evaluated with regard to validity in accordance with the recommendations and criteria provided in the professional manual. Negativity scale raw score of 6 or higher (maximum score is 10), Infrequency scale raw score of 3 or higher (maximum score is 5), and Inconsistency scale raw score of 8 or higher (maximum score is 10) indicate possible invalid protocols [48]. The inconsistency scale was not elevated for any of the participants. The Negativity scale was minimally elevated for one ADHD patient and two ADHD informants, reflecting that the participant answered 6–7 selected items in an unusually negative manner. The Infrequency scale was minimally elevated for five healthy controls with unusual responses to 3 out of 5 items comprising the scale (i.e., responded “never” for “I get tired,” “I make mistakes,” and “I get annoyed”). Each of these eight BRIEF-A protocols was inspected at both individual item level and at scale level. Because none of the protocols had an elevated score on more than one validity scale, and there were no highly atypical responses, or other indication of compromised validity we chose to include the eight protocols in the subsequent statistical analyses.

### Procedure

Prior to being scheduled for cognitive testing, the participants had been instructed to forgo taking any medication, nicotine or alcohol as the T.O.V.A. is sensitive to the therapeutic effects of stimulants [62]. The morning of the test session, participants were questioned about food intake, last night sleep, alcohol/nicotine use, and other drugs or conditions which could influence their cognitive performance. In order to increase the probability of obtaining valid results, participants were informed about the importance of providing accurate information on questionnaires and to give their best effort on the neuropsychological test.

At the time of cognitive testing, ADHD patients and healthy controls filled out the BRIEF-A and ASR self-report. For the patients, one close relative, friend or colleague completed the BRIEF-A and the ASEBA informant report. For all participants, the T.O.V.A. was done in the morning, in a room without environmental distractions. It was individually administered in accordance with the general task requirements. Standardised instruction was given verbally by a trained test administrator for the practice session and for the T.O.V.A. The participants were told that the test measures the ability to pay attention, that two different kinds of squares would flash on the computer screen, and that the squares would differ only in that one would have a small hole near the top and one would have a small hole near the bottom (Fig. 1). They were instructed to respond as quickly as possible to the Go signals, and to refrain from responding to the NoGo signals. Participants were told to hold the microswitch in their writing hand with their thumb resting lightly on the response button. They underwent a formal practice session prior to the actual test. The test administrator, who was present during the whole session, ensured that the participant understood the instructions, discriminated between the Go and NoGo signals, and was able to press the microswitch to the Go signals.

### Statistical analysis

Statistical Product and Service Solutions for Windows [63] was used for the statistical analyses. Preliminary assumption testing was performed to check for normality, linearity, univariate and multivariate outliers, homogeneity of variance-covariance matrices, and multicollinearity.

Except for the analysis of between-group differences in gender composition with chi square statistic, the demographic data were analyzed using independent samples t-test with Group (ADHD vs. healthy control) as between-subjects factor. Group differences in the four T.O.V.A. parameters Go signal reaction time, reaction time variability, omission errors, and NoGo signal commission errors were analyzed using repeated measures analysis of variance (ANOVA). The ANOVAs were defined by one repeated measure factor (Condition: 1^st^ vs. 2^nd^ half of the T.O.V.A., and additionally 1^st^ vs. 3^rd^ quarter for commission errors), and one between-subjects factor (Group: ADHD vs. control). Greenhouse-Geisser epsilon corrected p-values, along with uncorrected degrees of freedom are reported. Effect size was computed employing partial eta squared (η^2^). Significant interaction effects were decomposed using follow-up ANOVAs.

With regard to the BRIEF-A data, Levene's test of equality of error variances revealed that the assumption of equality of error variances of the dependent variables across groups were violated for all scales in the ADHD versus healthy control self-report analysis, and for a single scale (“Self-monitor”) in the ADHD self-report versus ADHD informant report analysis. Because of these findings, a more restrictive significance level, ≤.025, than the typical.05 level was set for all variables in the univariate F-tests [64].

One-way between-groups multivariate analyses of variance (MANOVA) were conducted to examine differences between a) ADHD and healthy control self-report scores, and b) ADHD self-report and ADHD informant report scores on the BRIEF-A. For each group comparison, one MANOVA included scores on the 9 scales as dependent variables.

Pearson product-moment correlation coefficients (two-tailed test) were used to test within-group relationships between T.O.V.A. standard scores and BRIEF-A scale T-scores, and between T.O.V.A. standard scores and T-scores on the scales Depression Problems and Anxiety Problems from the ASR-ASEBA. Average T.O.V.A. scores for the total of the task were subjected to correlation analyses. For the ADHD group, a follow-up analysis of significant correlations was performed using standard multiple regression analysis to test whether T.O.V.A. performance scores predicted scores on the BRIEF-A self- and informant report. We tested the overall fit of the model and the relative contribution of each of the predictors to the total variance explained.

Alpha for statistical analyses were set to p ≤.05, with the exception of an alpha level of ≤.025 for the MANOVAs conducted on BRIEF-A scale scores.

## Results

### Test of Variables of Attention

#### Group differences in T.O.V.A. scores

Table 2 shows the number of commission errors to NoGo stimuli as well as reaction time, reaction time variability, and the number of omission errors to detected Go signals for each group.

**Table 2:** Behavioral results on the T.O.V.A. for ADHD patients and healthy controls.

T.O.V.A.	ADHD (n = 36)		Control (n = 35)	
	Mean	SD	Mean	SD
**Go signals**				
*RT (ms)*				
1^st^ half	416.3	65.9	382.8	44.6
2^nd^ half	357.7	87.8	334.8	53.6
Total	371.5	79.5	345.9	49.2
*RT variability (ms)*				
1^st^ half	91.1	41.2	71.0	24.5
2^nd^ half	108.3	46.9	79.1	23.9
Total	110.9	44.4	81.6	22.3
*Omission errors*				
1^st^ half	0.7	0.9	0.2	0.7
2^nd^ half	4.4	8.2	1.6	3.4
Total	5.1	8.8	1.8	3.9
**NoGo signals**				
*Commission errors*				
1^st^ half	2.2	5.7	0.6	0.7
2^nd^ half	11.6	13.3	7.5	6.3
Total	13.8	16.3	8.1	6.6

Group mean raw scores and standard deviations (SD) are reported. Note. Omission and Commission errors  =  Number of errors.

*Go signal reaction time and reaction time variability:* There was a significant main effect of condition (1^st^ vs. 2^nd^ half) (*F*(1, 69)  = 84.94, p<.001, η2 = .55) reflecting that reaction times to detected Go signals were longer in the 1^st^ compared with the 2^nd^ half of the T.O.V.A. across groups. A marginally significant effect of group (*F*(1, 69)  = 3.86, p = .053, η2 = .05) was consistent with the tendency for generally less speedy reactions in the ADHD group relative to the control group. For reaction time variability a significant main effect of condition (*F*(1, 69)  = 10.36, p = .002, η2 = .13) was found, which reflected that reaction times were less consistent in the 2^nd^ compared with the 1^st^ half of the T.O.V.A. for both groups.

However, the ADHD group had significantly greater reaction time variability relative to the control group across conditions (*F*(1, 69)  = 10.78, p = .002, η2 = .14). There were no significant condition x group interactions for reaction time (*F*(1, 69)  = .832, p = .365, η2 = .01), or reaction time variability (*F*(1, 69)  = 1.36, p = .248, η2 = .02).

*Go signal omission errors*: The analyses showed significant main effects of condition (*F*(1, 69)  = 13.45, p<.001, η2 = .16) and group (*F*(1, 69)  = 4.11, p = .046, η2 = .06) reflecting that both groups made more omission errors in the 2^nd^ compared with the 1^st^ half of the T.O.V.A., but that the ADHD group made significantly more omission errors to Go signals across conditions compared to the controls. There was a non-significant trend for a condition x group interaction (*F*(1, 69)  = 2.94, p = .091, η2 = .04) in that ADHD patients tended to make more omission errors relative to controls in the 2^nd^ half of the task.

*NoGo signal commission errors*: The analyses revealed a significant main effect of condition (*F*(1, 69)  = 88.88, p<.001, η2 = .56), indicating more commission errors in the 2^nd^ (frequent Go signals) compared with the 1^st^ (infrequent Go signals) half of the T.O.V.A. across groups. There was a trend-level main effect of group (*F*(1, 69)  = 3.56, p = .063, η2 = .05) reflecting that the ADHD group tended to make more commission errors during the entire task compared to the controls. The condition x group interaction was not significant (*F*(1, 69)  = 1.96, p = .166, η2 = .03).

In order to test the hypothesis that adults with ADHD would be particularly prone to impulsive responding in task conditions with a high response demand, we conducted a separate repeated measure ANOVA that included the 1^st^ and 3^rd^ quarters of the task.

**Figure 2:**
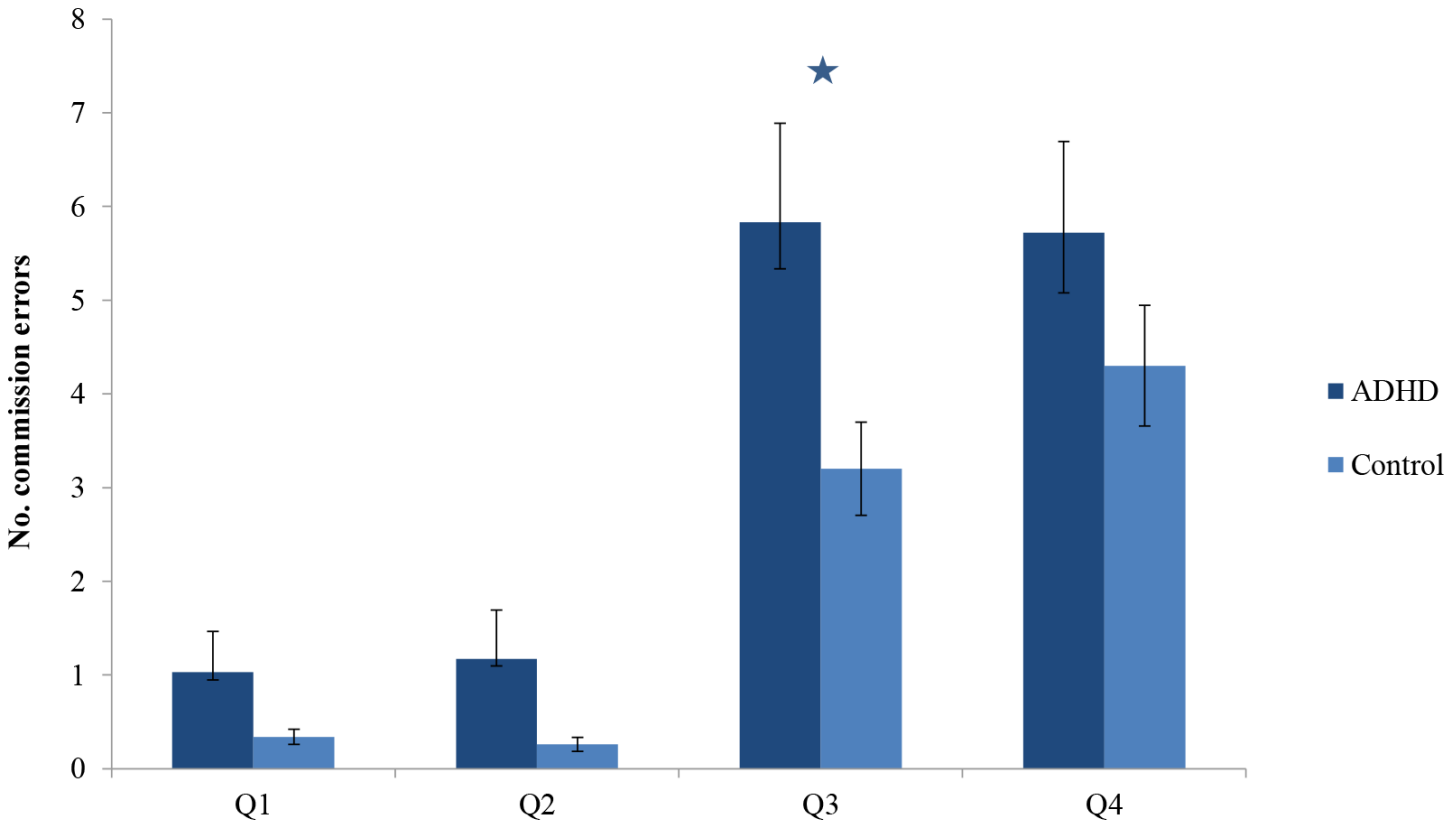
T.O.V.A. commission errors in quarters 1 to 4 for the ADHD patient group and the healthy control group.

A significant main effect of condition (*F*(1, 69)  = 65.31, p<.001, η2 = .49), was due to the sensitivity to increased response demand occurring from the 1^st^ to the 3^rd^ quarter across groups. A main effect of group (*F*(1, 69)  = 4.77, p = .032, η2 = .07) was modified by a significant condition x group interaction (*F*(1, 69)  = 4.07, p = .048, η2 = .06). A follow-up ANOVA revealed a significant group difference in commission errors in the 3^rd^ quarter (*F*(1, 69)  = 4.89, p = .030, η2 = .25) when there was a steep increase in the number of Go relative to NoGo signals (Fig. 2).

### Behavior Rating Inventory of Executive Function-Adult Version

#### Group differences in BRIEF-A scale scores

The multivariate test showed a statistically significant difference between the ADHD and healthy control self-reports on the combined dependent variables (*F*(9, 57)  = 22.43, p<.001; Wilks' Lambda  = .22; η2 = .78). When the results for the dependent variables were examined separately, all scales showed highly significant (all p's <.001) group differences (Table 3), reflecting that the ADHD group generally reported a greater degree of executive difficulties compared to controls. Effect sizes ranged from.34 for Organization of Materials to.73 for Working Memory.

**Table 3:** BRIEF-A and ASEBA self- and informant report for ADHD patients, and self-report for healthy controls.

BRIEF-A and ASEBA DSM Scales	ADHD Self-report		Control Self-report		ADHD Informant report		ADHD Self- vs. Control Self-report		ADHD Self- vs. ADHD Informant report	
	Mean	SD	Mean	SD	Mean	SD	p	η^2^	p	η^2^
**Metacognition**										
Initiate	68.1	13.4	42.7	4.5	59.5	12.2	.001	.617	.007	.104
Working Memory	75.0	11.8	45.2	5.4	64.2	13.6	.001	.725	.001	.156
Plan/Organize	66.9	11.9	44.6	4.7	58.5	12.4	.001	.600	.006	.109
Task Monitor	65.0	14.2	44.2	7.9	57.0	13.1	.001	.425	.019	.080
Organization of Materials	59.5	13.2	44.7	6.4	55.2	13.2	.001	.337	.181	.027
**Behavioral Regulation**										
Inhibit	67.4	12.2	43.1	5.9	58.8	12.4	.001	.615	.005	.112
Shift	63.2	12.2	42.2	4.3	54.6	11.0	.001	.569	.003	.124
Emotional Control	64.3	12.4	42.2	5.2	57.2	9.7	.001	.472	.011	.095
**ASEBA DSM**										
Depression Problems	67.7	9.1	50.8	2.0	63.8	10.4	.001	2.608		
Anxiety Problems	59.6	7.3	50.4	0.9	51.4	2.2	.001	1.818		

Group mean T-scores and standard deviations (SD) for each scale are reported along with p-values and effect sizes (η^2^) for group comparisons. Note. The empty cells for ADHD Self- vs. ADHD Informant report are due to lack of Informant report data on the ASEBA DSM scales.

For the comparison of ADHD self- and informant reports, the overall multivariate test was not significant (*F*(9, 58)  = 1.45, p = .157; Wilks' Lambda  = .81; η2 = .19). The omnibus univariate tests were, however, significant (p's <.02) for all scales with the exception of Organization of Materials. Inspection of the mean T scores (Table 3) showed that scores for ADHD self-report were larger than for ADHD informant report. For the significant effects, effect sizes ranged from.08 for Task Monitor to.16 for Working Memory.

##### Comparison of BRIEF-A scores to US normative cut-off values

As shown in Table 2, ADHD self-report resulted in 4 (Initiate, Working Memory, Plan/Organize, Task Monitor) of the 5 scales comprising the Metacognition Index being clinically elevated (defined as T score ≥65) according to the norms derived from the BRIEF-A normative sample [48]. The ADHD group had clinical elevation on 1 (Inhibit) of the 4 scales comprising the Behavioral Regulation Index. Both the Metacognition Index (mean T score  = 69.6; SD  = 13.2), and the Behavioral Regulation Index (mean T score  = 67.7; SD  = 12.9) were in the clinically elevated range.

Mean scale scores for ADHD informant report did not reach the clinically significant cut-off for any scale nor for the Metacognition Index (mean T score  = 59.8; SD  = 12.9) or the Behavioral Regulation Index (mean T score  = 57.2; SD  = 9.9). The healthy control self-report scores were without exception in the normal range for the scales as well as the Metacognition Index (mean T score  = 43.3; SD  = 5.0), and the Behavioral Regulation Index (mean T score  = 41.1; SD  = 4.7).

### Relation of T.O.V.A. performance to ratings on the BRIEF-A and ASEBA scales

Table 4 shows the relation of T.O.V.A. performance measures to BRIEF-A and ASEBA DSM scale scores for the ADHD group (self- and informant report) and the control group (self-report). All significant correlations were negative, indicating that poorer scores on the T.O.V.A. were associated with a greater degree of executive function difficulties reported on the BRIEF-A.

**Table 4:** Correlations of T.O.V.A. performance with BRIEF-A and ASEBA DSM self-report for the ADHD group and the healthy control group, and of the ADHD patients' T.O.V.A. performance with BRIEF-A and ASEBA DSM informant-report for patients.

BRIEF-A and ASEBA DSM scales	ADHD self-report				Control self-report				ADHD informant report			
	RT	RTvar	OE	CE	RT	RTvar	OE	CE	RT	RTvar	OE	CE
**BRIEF-A Metacognition**												
Initiate	−.07	−.19	−.40*	−.14	−.24	.22	−.02	.03	.05	−.13	−.17	−.24
Working Memory	−.07	−.06	−.32	.05	−.18	−.19	.14	.05	.13	.04	−.10	−.16
Plan/Organize	.06	−.19	−.34*	−.21	−.27	−.33	.18	−.04	.14	−.07	−.09	−.32
Task Monitor	.05	−.15	−.31	−.19	−.27	−.33	−.06	.05	.13	−.10	−.21	−.40*
Organization of Materials	.02	−.35*	−.54**	−.45**	−.12	−.06	−.03	−.18	.01	−.35*	−.40*	−.54**
**Behavioral Regulation**												
Inhibit	−.20	−.30	−.33	−.17	.23	.07	.04	.02	.02	−.12	−.16	−.06
Shift	.01	−.01	−.21	−.02	−.33	−.26	−.02	.09	.01	−.05	−.05	−.19
Emotional Control	−.23	−.17	−.34	.08	−.10	−.15	−.01	−.02	−.12	−.14	−.13	−.15
Self-Monitor	−.17	−.17	−.31	.04	.09	−.10	.06	−.01	−.17	−.29	−.29	−.16
**ASEBA DSM**												
Depressive Problems	.18	.11	−.05	.07	.04	−.10	−.01	.10	.16	.05	−.12	−.09
Anxiety Problems	.07	.02	−.21	−.02	.22	.30	.23	.25	.11	−.17	−.06	−.11

Notes. RT  =  Reaction time; RTvar  =  Reaction time variability; OE  =  Omission errors; CE  =  Commission errors;

* = p<.05;

** = p<.01.

*ADHD self- and informant report*: There were significant correlations between Go signal omission errors and *self-reported* scores on the BRIEF-A scales Initiate (*r*(33)  = −.40, p  = .019), Plan/Organize (*r*(33)  = −.34, p = .047), as well as between Organization of Materials and three out of four T.O.V.A. variables, namely omission errors (*r*(33)  = −.54, p = .001), commission errors (*r*(33)  = −.45, p = .007), and reaction time variability (*r*(33)  = −.35, p = .042).

The same pattern of associations involving the BRIEF-A Organization of Materials scale as seen for *self-report* was also observed for *informant report*: omission errors (*r*(32)  = −.40, p = .019), commission errors (*r*(32)  = −.54, p = .001), and reaction time variability (*r*(32)  = −.35, p = .046). There was also a significant correlation between commission errors and the Task Monitor scale scores (*r*(32)  = −.40, p = .020).

Due to the significant group by condition interaction for commission error in the T.O.V.A., we conducted an additional analysis to explore whether commission error in the 3^rd^ quarter had a stronger relationship to BRIEF-A scores than the total commission error score. There were no significant correlations between *ADHD self-report* and commission error in the 3^rd^ quarter, but *ADHD informant report* showed a significant correlation between 3^rd^ quarter commission error and the Organization of Materials scale (*r*(32)  = −.35, p = .041). However, the association was weaker than for the total commission error score (*r*(32)  = −.54, p = .001).

There were no significant correlations between the T.O.V.A. measures and the ASEBA Depression Problems or Anxiety Problems scales.

*Healthy control self-report*: There were no significant correlations of the T.O.V.A. variables with the BRIEF-A or the ASEBA DSM scale scores (Table 4).

Table 5 shows the relationships between BRIEF-A and ASEBA DSM scale scores for the ADHD group (self- and informant report) and the control group (self-report). Almost all significant correlations were positive, indicating that more severe symptoms endorsed on the ASEBA DSM mood scales were associated with a greater degree of executive function difficulties reported on the BRIEF-A.

**Table 5:** Correlations between ASEBA and BRIEF-A self-report for the ADHD group and the healthy control group, and between the ADHD patients' ASEBA and BRIEF-A informant-report.

BRIEF-A scales	ASEBA DSM					
	ADHD self-report		Control self-report		ADHD informant report	
	Depressive	Anxiety	Depressive	Anxiety	Depressive	Anxiety
**Metacognition**						
Initiate	.47**	.47**	.43*	.13	.56**	.26
Working Memory	.31	.38*	.35*	−.01	.51**	.37*
Plan/Organize	.26	.28	.37*	−.01	.53**	.31
Task Monitor	.28	.30	.58**	−.02	.50**	.28
Organization of Materials	.15	.05	.11	−.39*	.29	.21
**Behavioral Regulation**						
Inhibit	.10	.19	.50**	.09	.34	.30
Shift	.31	.45**	.09	−.14	.40*	.35*
Emotional Control	.10	.17	.59**	−.03	.35*	.23
Self-Monitor	.20	.09	.58**	.08	.34	.19

Notes. * = p<.05; ** = p<.01.

*ADHD self- and informant report*: There were significant correlations between *self-reported* scores on the ASR DSM Depression Problems and the BRIEF-A Initiate scale (*r*(32)  = .47, p = .005), and between the ASR DSM Anxiety Problems and the BRIEF-A scales Initiate (*r*(32)  = .47, p = .005), Working Memory (*r*(32)  = .38, p = .029), and Shift (*r*(32)  = .45, p = .007).

There were also significant correlations between *informant reported* scores on the ASEBA DSM Depression Problems and the BRIEF-A scales Initiate (*r*(32)  = .56, p = .001), Working Memory (*r*(32)  = .51, p = .002), Plan/Organize (*r*(32)  = .53, p = .001), Task Monitor (*r*(32)  = .50, p = .003), Shift (*r*(32)  = .40, p = .018), and Emotional Control (*r*(32)  = .35, p = .040). The analysis revealed additional significant correlations between the ASEBA DSM Anxiety Problems and the BRIEF-A scales Working Memory (*r*(32)  = .37, p = .034), and Shift (*r*(32)  = .35, p = .043).

*Healthy control self-report*: There were significant correlations between *self*-*reported* scores on the ASR DSM Depression Problems and the BRIEF-A scales Initiate (*r*(30)  = .43, p = .015), Working Memory (*r*(30)  = .35, p = .048), Plan/Organize (*r*(30)  = .36, p = .040), Task Monitor (*r*(30)  = .58, p = .001), Inhibit (*r*(30)  = .50, p = .004), Emotional Control (*r*(30)  = .59, p = .001), and Self-Monitor (*r*(30)  = .58, p.001). A significant negative correlation between the ASEBA DSM Anxiety Problems and the Organization of Materials scale (*r*(30)  =  −.39, p = .028) was also observed (Table 5).

Due to the significant correlations between the ADHD self- and informant report scores on the BRIEF-A Organization of Materials scale and results on the T.O.V.A., standard multiple regression analyses were conducted to explore if the T.O.V.A. variables reaction time variability, omission errors, and commission errors significantly predicted ratings of executive function difficulties.

The full model containing all three predictors explained 37% of the variance in Organization of Materials for the ADHD *self-report* (*R* ^2^ = .37; p*<*.001), and 35% for the ADHD *informant report* (*R* ^2^ = .35; p*<*.001) (Table 6). Of the three independent variables in the model, omission errors made the strongest unique contribution (*β* = −.52; p = .009) for the ADHD *self-report*, whereas commission errors made the strongest unique contribution (*β* = −.52; p = .008) for the ADHD *informant report* when the variance explained by the other variables was controlled for.

**Table 6:** Summary of multiple regression models predicting ADHD self- and informant report scores on the BRIEF-A Organization of Materials scale from the patients' T.O.V.A. reaction time variability, omission- and commission errors.

Model	Beta	t	R^2^	Adj R^2^	SE
**ADHD self-report**					
Organization of Materials		10.82**	.37	.31	10.98
** Predictors**	.20	.94			
RT variability					
Omission errors	−.52	−2.78**			
Commission errors	−.35	−1.99			
**ADHD informant report**					
Organization of Materials		9.92**	.35	.29	11.33
** Predictors**	.15	.68			
RT variability					
Omission errors	−.31	−1.60			
Commission errors	−.52	−2.87**			

Notes. RT  =  reaction time; Adj R^2^ =  Adjusted R^2^; SE  =  standard error;

* = p<.05;

** = p<.01.

## Discussion

The main aim of the current study was to examine attention and motor response control in treatment-naive adults with newly diagnosed ADHD. Using a Go/NoGo paradigm (T.O.V.A.) requiring sustained visual attention we found that in comparison to demographically matched healthy controls, the ADHD group had performance deficits that indicated both inattention and impulsive responding. Importantly, reduced task performance was significantly associated with higher self- and informant ratings of selective aspects of cognitive executive difficulties on the BRIEF-A. In the following, the findings will be discussed in relation to previous studies and implications for clinical evaluation of adult ADHD.

### Performance on the Test of Variables of Attention

Reaction times to correctly detected Go signals in the T.O.V.A. were significantly longer for both groups in the first half of the task where Go signals occurred infrequently, compared to the higher response demand of the second half. Task event rate has been seen as a key probe of central nervous system activation processes, and has been found to differ between ADHD and healthy children. Metin and coworkers found a disproportionate slowing of reaction times in ADHD children relative to controls in the condition of slow event rates in a Go/NoGo task [65]. They interpreted this to be due to underactivation resulting from a failure to adjust activation level according to the demands of long and boring tasks. In our study, however, the adult ADHD group had a tendency for less speedy reaction times compared to the healthy group, that was independent of the ratio of Go to NoGo signals.

Also, both groups showed greater reaction time variability in the second half of the T.O.V.A. when the task required more frequent responding. However, the ADHD group had more inconsistent reaction times compared to the healthy controls for the total duration of the task, indicating difficulties with sustained attention. Previous ADHD literature has argued that inconsistent response time can be seen as a behavioral marker of inattention in ADHD [66], [67] that may persist into adulthood. In a large-scale study of adult ADHD, it was found that variability in reaction time during a CPT paradigm significantly differentiated the ADHD group from comparison participants [5]. Inconsistent performance on tasks demanding sustained attention in both children and adults with ADHD [5], [68], [69] has been explained by lapses in attention due to an inability to maintain alertness and directed focus during prolonged mental activity [70]. The processes underlying variable response times are thought to be related to the propensity to be distracted by external stimuli [46]. The results of the present study are in accordance with the proposition that intra-individual response time variability is a core feature of ADHD also for adults [71]. Inconsistent responding has been suggested as a useful endophenotype for childhood ADHD [72], [73]. Our results indicate that this may also hold true for adult ADHD.

Accompanying the prolonged and more variable reaction times to detected Go signals, adults with ADHD made more Go signal omission errors during the entire T.O.V.A. than healthy controls. This finding is in agreement with other studies employing CPTs in studies of adult ADHD [45], [46], [74], [75]. Altogether, the behavioral response pattern of inconsistent reaction speed and increased rate of omission errors to Go signals in the T.O.V.A. suggests pronounced inattention in adult ADHD.

Deficient inhibitory control in adult ADHD has been reported previously [22], [34], [74], [76], [77], particularly in contexts containing an established prepotent response bias [22]. In the present study, the number of commission errors to NoGo signals for the entire T.O.V.A. differed only at trend-level between the ADHD patients and the healthy controls. Both groups made significantly more commission errors under the most taxing attentional conditions, i.e., when the frequency of Go signals changed from low to high in the second half of the task. However, an analysis that specifically tested the effect of the initial phase of low (1^st^ quarter) versus the initial phase of high (3^rd^ quarter) motor response demand demonstrated significant condition-dependent differences between the groups. The ADHD group made significantly more commission errors compared to controls when the response demand increased. This shows that experimental manipulation of response prepotency was effective in evoking response inhibition difficulties in the adult ADHD group. The reduction in motor response control, but not in other performance parameter, when the Go/NoGo ratio changed from low to high response demands might reflect dysfunction in specific aspects of neural networks. Condition-dependent impairment in motor inhibition on a laboratory task may be a useful proxy for the relative context-dependence of real-life impulsive behavior in patients with ADHD [78].

A possible explanation for the diversity of executive problems in ADHD has been proposed in a model of inhibitory control [79]–[83]. Barkley suggested that inhibitory control deficit is related to difficulties in the broader domain of executive function, and is ultimately reflected in impaired behavioral expressions [80]. Although this view represented a parsimonious account of ADHD, there has been a transition from models positing a single core deficit to multiple-deficit models for conceptualizing the neuropsychology of the disorder [84]. As part of this process, theoretical models are beginning to account for the neuropsychological heterogeneity of ADHD [3], [20], [66], [85], [86], and other multiple-deficit models suggest that ADHD is attributable to the additive or interactive effects of dysfunction in multiple neural networks [87], [88]. Theoretical models postulating multiple cognitive deficits and associated large-scale neural networks dysfunction are likely needed to explain the diversity in behavioral expression of brain dysfunction in ADHD. To explore this model we also examined whether adults with ADHD experienced problems of executive function in their daily lives, and whether any perceived difficulties were associated with performance on a laboratory-based task demanding sustained attention and dynamic response control.

### Self- and informant reported executive function

The examination of group differences in subjective concerns related to executive functioning revealed that the ADHD patients exhibited significantly higher scores on both the summary dependent BRIEF-A variable computed in the multivariate statistical analysis, as well as on every single scale, relative to the healthy controls. The scores were 2 SDs or more above the scores of the healthy control group on most scales. Also, compared to the US normative values [48], the ADHD patients had clinically elevated scores on 5 of the 9 scales, of which 4 belonged to the Metacognition Index and 1 (Inhibit) to the Behavioral Regulation Index. The results show that the ADHD group reported experiencing significant impairment in everyday competence with regard to cognitive executive control in particular, but also with control of impulsive behavior.

The comparison of ADHD self- and informant report did not show a significant group difference on the BRIEF-A summary variable. However, the analyses of the BRIEF-A scales separately revealed significant group differences on all but one scale. The ADHD informant scores did not exceed the US normative cut-off score, indicating that the informants viewed the patient's behavior as less burdensome than the patients themselves. Interestingly, worries concerning working memory had the strongest effect size whether ADHD self-report was compared with informant report or healthy control self-report.

To our knowledge, few studies have used the BRIEF-A to assess executive function in adult ADHD. In a study investigating the factor structure of the BRIEF-A, the adult ADHD group reported significantly greater difficulties than healthy controls on scales encompassing the Metacognition Index, whereas scales belonging to the Behavioral Regulation Index showed a trend-level group difference [89]. This is in accordance with the present study which found that a greater number of clinically elevated subscales in the ADHD group belonged to the Metacognition Index than the Behavioral Regulation Index when US norms were used for comparison.

In two studies using the BRIEF-A to measure effects of medication on executive function, the results showed clinically elevated baseline scores on several scales [90], [91]. Moreover, an unmedicated adult ADHD sample (n = 27) referred to in the BRIEF-A professional manual had clinically elevated scores on all 5 scales comprising the Metacognition Index (mean T score  = 77.7), and 2 scales (Inhibit and Self-Monitor) of the Behavioral Regulation Index (mean T score  = 68.1) [48]. The patterns of results suggest similar worries about executive function in the latter and in our ADHD cohort, but a slightly lower level of concern related to executive function in our ADHD group. Altogether, the studies suggest that adult ADHD is associated with significant self-reported impairment across a wide range of executive functions. A next question is whether measures of executive control in an attention demanding task are related to experienced executive difficulties and to current mood.

### T.O.V.A. performance in relation to self- and informant reported executive function and mood

The healthy control group showed no significant associations between the BRIEF-A or the ASR DSM scales and results on the T.O.V.A. A lack of significant correlations between task performance and scores on the ASR DSM mood scales were also seen for the ADHD group, but there were moderate negative correlations between T.O.V.A. results and a subset of BRIEF-A scales belonging to the Metacognition Index. The significant correlations between reaction time variability and error rates for both ADHD self-report and informant report on the Organization of Materials scale may suggest a particular relevance of the attention and behavior regulation problems detected on the T.O.V.A. to organizational difficulties in everyday situations. Notably, the follow-up multiple regression analyses revealed that T.O.V.A. performance measures qualified as significant predictors of both self- and informant reported problems with organizing and keeping track of personal belongings often associated with impairment at work and in home life.

### Self- and informant reported executive function in relation to current mood state

There were significant associations between both self- and informant reported BRIEF-A and ASEBA DSM mood scores. A moderate negative correlation between the BRIEF-A subscale Organization of Materials and ASEBA DSM Anxiety Problems for the healthy control group self-report indicated that a low level of anxiety was associated with a greater level of perceived organizational difficulties.

The predominantly moderate positive correlations between BRIEF-A and ASEBA DSM scores for both self- and informant report for both groups indicate that increased emotional distress was associated with an increased level of perceived executive difficulties in everyday life. This is in accordance with the growing evidence for a brain-based association between executive function and mood [52]. A study by Roth and colleagues employing confirmatory factor analysis showed a differential pattern of correlations between self-reported mood and the factor scores on BRIEF-A in young adult ADHD patients [89]. In a three-factor BRIEF-A model they found that self-reported depressed mood was highly correlated with an Emotional Regulation factor [89]. Altogether, our and previous studies indicate that current mood interact with executive function and should be taken into account when evaluating ratings of perceived executive functioning in everyday life.

### Limitations and strengths of the study

The clinical nature of the study in some ways limits the ability to generalize the results to the total adult ADHD population. The study included adults who had mainly been referred from psychiatric outpatient clinics, and reported major problems in several aspects of living such as adaptation to the requirements for education and work life. The consequence is that the results may not be representative for adults with ADHD who are able to compensate for some of their deficits and therefore have a better adjustment to the demands of adulthood.

The clinical group studied is known to have high intra- and inter-individual variability in both cognitive function and in overt behavioral symptoms [7], [22]. We therefore aimed towards rigorous experimental control and careful matching of the two comparison groups on the factors age and education as these are potential moderator variables for cognitive function [92]. Aiming to control for factors that might influence the test results, each participant was screened for sensory-perceptual deficits and was carefully questioned before testing about for example sleep, medications, and intake of substances potentially having an effect on the central nervous system. Another asset is that all patients were treatment-naive. A history of stimulant treatment might have presented a confounding variable as treatment-related “normalization” of cognitive function, and volumes of brain regions supporting executive control have been reported in children and adolescents with ADHD [93].

We carefully assessed the validity of the scores obtained from both the BRIEF-A and the T.O.V.A., and related the findings to information collected in clinical interview. Thus we followed Roth and colleagues advice to base validity assessments on data from multiple sources i.e., from validity scales to clinical information [48]. Our approach to validity testing is supported by a recent study showing that there were only small to modest growth effects when the number of standardized validity tests was increased beyond two [94].

The comparison of results on the BRIEF-A with US normative data may represent a limitation. Similarly to another Norwegian study by Løvstad and colleagues [51], we found that the mean T-score for the healthy controls was 0.5–1 SD below the US normative sample mean T-score. This suggests that T-scores below the clinical cut-off of 65 may provide clinically important information to clinicians assessing patients in Norway. In the present study, the use of a well-matched healthy control group from the same geographic area as the patients reduced the potential limitations in using US norms.

Few studies have focused on adult ADHD and impairment of executive functions after the early twenties [10], [95], [96]. Our study adds to this literature in showing that both task performance and subjective ratings of executive problems continue to be associated with functional impairment into mid-life. The sample size in this study was relatively small, but similar to previous studies of adult ADHD [22], [97]. Nevertheless, the small sample size limits the ability to make proper comparisons between ADHD patients with different comorbidities related to psychological health.

### Summary and clinical implications

The present study combined behavioral measures of attentional control and response inhibition with self- and informant report measures of executive function. The results showed that a clinical ADHD group reporting a high degree of executive difficulty in daily living was significantly impaired on a laboratory task demanding sustained attention and response inhibition. Moreover, performance on the cognitive task significantly predicted perceived organizational difficulties. Altogether, the findings indicate that in a clinical cohort of treatment-naive adults with ADHD-combined type, the problems with inattention and inhibitory control are largely similar to those reported in childhood ADHD.

Although this study demonstrated impaired performance on the T.O.V.A., along with self- and informant reported problems in executive function, we have not fully characterized adult ADHD cognitively and behaviorally, and cannot recommend use of single performance measures or rating scales for diagnostic purposes. However, because the T.O.V.A. measures central aspects of attention and impulsivity under controlled conditions, it can give a useful contribution to a thorough functional assessment of ADHD. The BRIEF-A can serve a similar purpose. With its nine theoretically and empirically derived clinical scales, important aspects of executive functioning from both self- and informant report perspectives can be evaluated. Importantly, Gioia and coworkers recommended the BRIEF not to be used as a diagnostic tool by itself, but should best be used within the context of a broad clinical evaluation [98]. Our findings indicate that the T.O.V.A. and the BRIEF-A represent measures of executive control at different levels of analysis. This view is in line with the results of a recent review by Toplak and colleagues. The authors concluded that whereas performance-based measures of executive function give information on processing efficiency, ratings of everyday executive function provide an indication of the success in individual goal pursuit [99]. As shown in the present study, the T.O.V.A. and the BRIEF-A, complement each other in that they provide partly distinct and partly associated information on important characteristics of adult ADHD.
